# Synthesis of Pyridinic-Rich N, S Co-doped Carbon Quantum Dots as Effective Enzyme Mimics

**DOI:** 10.1186/s11671-017-2149-y

**Published:** 2017-05-25

**Authors:** Teng Liu, Zhi-wei Cui, Jun Zhou, Ying Wang, Zhi-gang Zou

**Affiliations:** 1 School of Chemistry and Chemical Engineering, Eco-materials and Renewable Energy Research Center (ERERC), Nanjing, China; 2 National Laboratory of Solid State Microstructures, Nanjing, China; 3 Jiangsu Key Laboratory for Nanotechnology, Kunshan Innovation Institute of Nanjing University, Nanjing, 210093 China

**Keywords:** Quantum dots, N-doping, Photoluminescence, Enzyme mimics catalysis

## Abstract

N and S co-doped carbon quantum dots (N, S-CQDs) with high N- and S-doping level were synthesized by microwave solid-phase pyrolysis within 50 s. Owing to the dominant pyridinic N injection into the conjugated framework, both high enzyme mimics catalytic activity and photoluminescence quantum yield are achieved simultaneously.

## Background

The carbon quantum dots (CQDs) which emerged as a novel zero-dimensional carbon material have received tremendous attention because of their high chemical stability, low cytotoxicity, and unique electronic nature and optical behaviors [1–3]. With active surface groups such as –OH and –CO_2_H, CQDs can be recombined with other organics or inorganics for various fantastic applications including bio-imaging [4, 5], optoelectronic devices, and photocatalysts for degradation of organic dyes or production of hydrogen from water splitting [6–8]. Very recently, both experimental and theoretical results confirmed that heteroatom doping was an effective method to improve the electronic and optical properties of CQDs [9, 10]. Among the novel composites, N-doped CQDs or nitrogen/sulfur co-doped CQDs (N, S-CQDs) demonstrated much high fluorescence quantum efficiency or photocatalytic activity than that of pristine one [11, 12]. Also, the enhancement in performance of N-doped CQDs had shown a positive correlation to the nitrogen doping amount [13, 14]. Although these studies convincingly certify that N-doping strikingly influences the properties of CQDs, however, there are scarce reports on the effective hetero-doping methods for CQDs. Suffered from the high solubility of inorganic precursor of the dopant, conventional hydrothermal carbonization routes would lead to large amount of dopants remaining in the reaction solution and thus quite low N-doping amount in the final CQDs.

Herein, we reported synthesis of nitrogen-rich N, S co-doped carbon quantum dots (N, S-CQDs) by microwave-assisted approach within only 50 s. Citric acid (CA) was chosen as carbon source, and thiourea was used as not only a nitrogen and sulfur source but also a weak base. The nitrogen and sulfur concentration of N, S-CQDs reaches 12.8 and 7.2 wt%, respectively, which was about five and three times higher than reported for N-CQDs and N, S co-doped CQDs [11, 14].

## Methods

The N, S-CQDs were obtained in the following ways: the mixture of 0.42 g (2 mmol) of citric acid monohydrate and 0.46 g of (6 mmol) thiourea was put in a porcelain crucible and heated for 50 s in a microwave reactor (445 W). The obtained brownish yellow product was added into 30 mL of deionized water to form a yellow suspension and centrifuged at 9000 rpm for 20 min. Then, the supernatant was purified with a 0.22-μm filter membrane and dialyzed with deionized water through a dialysis membrane (retained molecular weight,1000 Da) for 24 h. Finally, the dialysate was further freeze-dried under vacuum. The pristine CQDs were synthesized from neat citric acid monohydrate, and the subsequent treatment process was the same with that of N, S CQDs.

The enzyme-mimic activity of N, S-CQDs for the decomposition of H_2_O_2_ was measured in a 30-mL buffer solution of citric acid–disodium hydrogen phosphate (pH ≈ 3.5, 35 °C) containing 1 μg mL^−1^ of N, S-CQDs and 8 × 10^−4^ M of tetramethylbenzidine (TMB) substrate. After 160 μL of H_2_O_2_ (30%) solution was added to the colorless buffer solution, the reaction starts and then takes the solution to measure the absorbance of the blue oxidation product of TMB at 652 nm each 2 min. Finally, the reaction rates of oxidation TMB were calculated. The reusability test of N, S-CQDs was performed in the reaction system containing 60-mL buffer solution of citric acid–disodium hydrogen phosphate and 2 μg ml^−1^ of N, S-CQDs as well as 5 × 10^−3^ M of TMB substrate. The reaction started as the addition of H_2_O_2_ solution (0.3%, 320 μL) into the mixed solution and took a small amount of solution to measure the absorbance at 652 nm after 1 h and the first cycle was finished. Then, 320 μL of fresh H_2_O_2_ (0.3%) solution was added to the reaction system for the next cycle. Other three-time cycle reactions were repeated in the same condition. The corresponding absorbance was calculated by subtracting the last absorbance.

The transmission electron microscope (TEM) and high-resolution transmission electron microscope (HRTEM) images were obtained on a JEM-2100 electron microscope with a high voltage (200 kV). The selected-area electron diffraction (SAED) was measured by FEITF20 (FEI high-resolution field-emission transmission electron microscopy) with a condition of 200 kV. The UV/vis absorption spectra were carried out with UV-3600 (Shimadzu UV-VIS-NIR Spectrophotometer). The fluorescence spectra were recorded on F-7000 (Hitachi Fluorescence Spectrometer) with the condition of 700 V. The fluorescence lifetime and FLQY were measured by the FM-4P-TCSPC (Horiba Jobin Yvon). The excitation and emission wavelengths are 358 and 436 nm, respectively. The X-ray powder diffractometer (XRD) were characterized by D8 Advance (Germany Bruker AXS Ltd.) using Cu K*α* with the condition of 40 kV and 40 mA. The Fourier transform infrared (FT-IR) spectra were carried out with Nicolet iS10 (Thermo Fisher Infrared Spectrometer). X-ray photoelectron spectrometer (XPS) was obtained on PHI 5000 Versa (UIVAC-PHI). TG-MS (thermogravimetric-mass spectrometry) is measured by Netzsch STA 449C with a heating rate of 10 K min^−1^ from 35 °C up to a final temperature of 450 °C under the N_2_ air (10%, air) flow.

## Results and Discussions

**Fig. 1:**
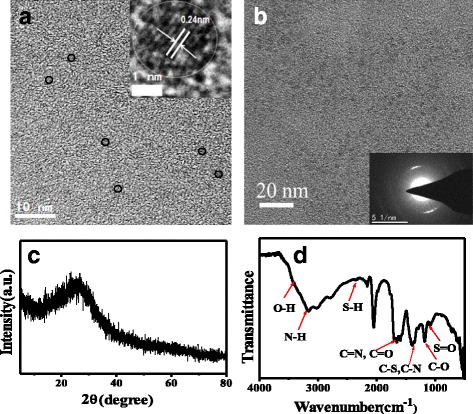
**a** TEM image of N, S-CQDs (the insert is HRTEM image). **b** SAED image of N, S-CQDs. **c** XRD and **d** FT-IR spectrum of N, S-CQDs

It can be seen from the TEM images (Fig. 1a) that the as-prepared N, S-CQDs are uniform and well-dispersed thin nanosheets with an average size of 2.0 nm in diameter. The inserted HRTEM image (Fig. 1a) shows very clear lattice fringe spacing of 0.24 nm consistent with the (1120) facet of graphene, indicating the crystalline cores of N, S-CQDs which might be composed of graphitic *sp* ^2^ carbon atoms [15, 16]. The inserted SAED image (Fig. 1b) shows the N, S-CQDs are crystalline with a lattice fringe of 0.312 nm corresponding to the reported graphic N-CQDs [13]. This *d* value agrees well with the interplanar spacing of (002) diffraction facets of reported N, S co-doping CQDs with graphitic structure [11]. The XRD pattern of N, S-CQDs illustrates a single broad peak centered at 2*θ* value of around 25.5° that was assigned to the diffraction peak of graphene (Fig. 1c), corresponding to the interlayer spacing of 0.33 nm [17]. However, g-CNQDs and β-C_3_N_4_ synthesized by urea with sodium citrate or citric acid have been reported [18, 19]. Different with our samples, the g-CNQDs have two of the characteristic peaks at 27.4° and 13.1° in the XRD. The strong peak at 27.4° represents the characteristic interplanar stacking of aromatic systems, indexed for graphitic carbon nitride as the (002) peak, and the weak diffraction peak at 13.1° corresponds to an interplanar structural packing motif indexed as the (100) peak. The g-CNQDs were synthesized with high molar ratio (6:1) of N/C precursor (urea to sodium citrate) [18]. In addition, more N injected into the core to form carbon nitride dots with a longer thermal treatment time reach to 60 min. But the heating time for microwave solid-phase pyrolysis of our N, S-CQD sample is only 50 s. Different with our samples, the volume of the mixture of urea and citric acid solution is boiled up to a point of 100 °C and obtained β-carbon nitride nanocrystalline. In a conclusion, we speculate that low mole ratio of N/C precursor, short reaction time, and relatively higher temperature may lead to a graphitic carbon structure. Compared to that of free-doping CQDs [20], the (002) diffraction peak of our N, S-CQD sample shifts from 23° to a higher angle of 25.5°, implying a decrease of interlayer spacing. Strong interplanar electronic stacking interactions between the graphene-like layers of N, S-CQDs. Possessing stronger electronegativity than carbon atom, the hetero-doping of large amount of nitrogen and sulfur atoms in the conjugated carbon framework would cause an increase in the electronic density of the whole conjugated carbon framework, and thus, the interplanar distance shortened [21, 22].

**Fig. 2:**
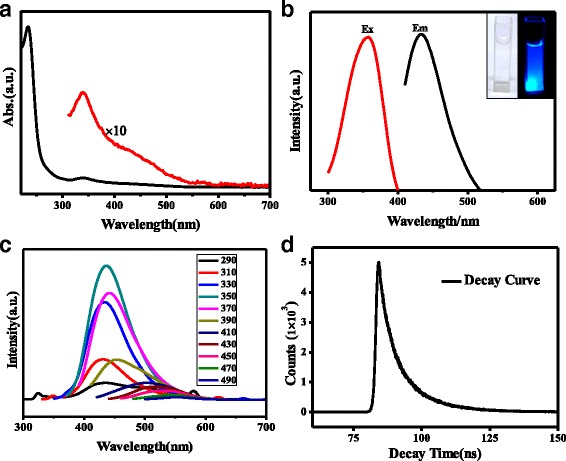
**a** The UV/vis of N, S-CQDs. **b** The PL of N, S-CQDs and the *inset* is the images of N, S-CQDs under ambient light and 365 nm irradiation. **c** The PL spectra of N, S-CQDs with different excitation wavelengths. **d** Photoluminescence intensity decay curves at the excitation light of 358 nm

The FT-IR spectrum (Fig. 1d) confirms various surface groups of N, S-CQDs. The bands at 3163cm^−1^ with a shoulder at 3416 cm^−1^ in 3000–3500 cm^−1^ range represent N–H and O–H stretching vibrations, respectively [11]. These considerable amino, hydroxyl hydrophilic groups could enable N, S-CQDs superior hydrophilicity [23]. The triple peaks that appear at around 1582, 1656, and 1704 cm^−1^ can be assigned to the different characteristic vibration bonds, respectively. The peaks at around 1704 cm^−1^ are stretch vibration of C=O carboxylic groups and C=N bonds [24], and the other two peaks at 1656 and 1582 cm^−1^ are the characteristic vibration of amide groups stretching C=O (amide I) and in-plane bending of N–H bond (amide II) [24, 25]. The peaks at 1405 and 1345 cm^−1^ can be assigned to the vibration of C–S and C–N, respectively [17], while the bands at 1177 and 1084 cm^−1^ further confirm the existence of C–O and S=O bonds on N, S-CQDs [17, 23]. UV/vis absorption spectrum of N, S-CQDs depicts two clear absorption bands (Fig. 2a). The strong absorption band at 234 nm is ascribed to the π-π* electronic transition of aromatic conjugated system *sp* ^2^ domains [17], while the weak absorption peak at 340 nm is attributed to the n-π* transition of C=O bond [26]. It was noticed that the relative intensity of absorption peak at 234 nm is much stronger than that of the N, S-CQD samples synthesized by hydrothermal method [17, 26], suggesting the formation of more aromatic *sp* ^2^ domains with N-doping into conjugated core system as pyridinic N. Besides, a broad shoulder at around 430 nm overlapped by the peak at 340 nm stems from many kinds of surface state transitions [26].

The photoluminescence (PL) spectra (Fig. 2b) illustrate that the N, S-CQDs have broad distribution of excitations. The maximum excitation wavelength is at 358 (emission wavelength 436 nm) owing to the 340 nm of absorption peak. It can be seen from the inset image in Fig. 2b that the colorless and transparent N, S-CQDs aqueous solution becomes bright blue under 365 nm UV irradiation. The solution of N, S-CQDs remained clear for 10 months without precipitations; this high stability of N, S-CQD particles is ascribe to their much small and uniform size as well as hydrophilic groups on the surface.

**Fig. 3:**
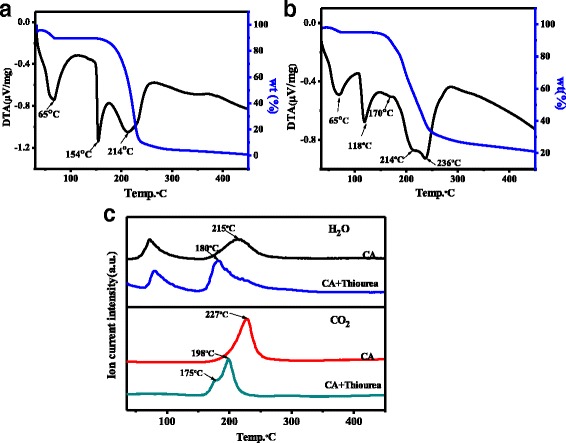
TG and DTA curves of **a** neat citric acid, **b** reactant mixture of citric acid and thiourea. **c** TG-Mass curves of neat CA and reactant mixture of citric acid and thiourea

The photoluminescence quantum yield (PLQY) of N, S-CQDs is calculated to be 23.6% under excitation at 358 nm, which is three times higher than that the reported for N-doped or N, S-CQDs [20, 23]. In contrast, the PLQY of pristine CQDs is only 1.15% that is far less than that of N, S-CQDs. It has been reported that the PLQY of CQDs was related to the N-doping amount in CQDs [17, 27], and thus, many works have attempted to increase the N-doping amount in CQDs by prolonging the reaction time even up to 19 h or raising the reaction temperature to 260 °C [11, 23]. However, the N-doping amount in the final solid samples was still less than 6%. In our work, high N-doping level of 12.5% is achieved on N, S-CQDs by an efficient solid-phase microwave-assisted way, wherein citric acid and thiourea molecules rapidly react and avoid sublimation. In addition, thiourea works as a weak base to accelerate the polymerization rate. Intriguingly, changing the ratio of thiourea and CA from 3:1 to 1:3 and 1:1, the PLQY of N, S-CQDs was slightly reduced to 7 and 2.1%, respectively. Moreover, increasing the reaction time up to 2 min merely obtained the bulk carbon. To further clarify the reaction mechanism, we acquired the thermogravimetric-differential thermal analyzer (TG-DTA) and TG-mass curves of neat CA and the reactant mixture of CA and thiourea. As displayed in Fig. 3a, there are three exothermic peaks that can be observed in the TG-DTA curves of neat CA; the first peak corresponds to the absorbing water including crystal water. The second sharp peak at 154 °C assigns to the melting heat release of CA crystalline, and the broad one centered at 214 °C relates to the intermolecular dehydration and carbonization. Whereas for the mixture of CA and thiourea, much changes on the latter two exothermic peaks can be observed on the TG-DTA spectrum (Fig. 3b), the second exothermic peak appears at a low temperature of 118 °C, indicating that the acid–base interaction between CA and thiourea results in a dramatic drop of 36 °C in the melting heat release step. In addition, the besides third exothermic peak that corresponded to the dehydration and carbonization derives two peaks at 214 and 236 °C, and a weak peak at 170 °C can be observed, implying that the addition of thiourea can advance the dehydration and carbonization process. Comparing the TG-mass spectra of the neat CA and the mixture of CA and thiourea (Fig. 3c), it can be found that the temperature maximum of H_2_O release peak decreases from 215 °C for the neat CA to 180 °C for the mixture of CA and thiourea. Similarly, the maximum of the CO_2_ release peak is at 227 °C for CA, but it shifts to low temperature and becomes a two-step release at 179 and 198 °C for the reactant mixture of CA and thiourea, respectively. This temperature decrease in the dehydration and carbonization well agrees with the TG-DTA results, implying different reaction approach for these two systems. For the neat CA, the intermolecular dehydration and carbonization simultaneously occurs at high temperature. While for the mixture of CA and thiourea, the intermolecular dehydration reacts firstly between the carboxyl groups of CA and the amino groups of thiourea, and then, the stepwise carbonization happens to form the carbon core of N, S-CQDs. Compared to the weak hydrogen bond interaction between the CA molecules, strong acid–base interaction between carboxyl groups and amino groups results in the significant decrease of dehydration temperature. Intriguingly, as displayed in Fig. 3c, the residual mass for the neat CA and the mixed reactant of CA and thiourea are 1 and 21 wt%, respectively, which indicates that the added thiourea can play a role as a weak base to lower the reaction temperature and avoid sublimation, thus enhances the N- and S-doping content in N, S-CQDs with high yield.

Figure 2c illustrates the emission spectra of N, S-CQDs with different excitation wavelengths. When the excitation wavelength changes from 290 to 370 nm, the peaks of emission at 440 nm show nearly no shift. The emission components are fairly constant in energy and most probably originate from absorption of n-π* transition at 340 nm. The excitation-independent emission property of CQDs has been studied by fitting the complex emission peaks to multiple Gaussian functions and deduced similar conclusion [26]. While when the excitation wavelength is varied from 390 to 490 nm, the PL emission spectrum exhibited a redshift as the increase of excitation wavelength, characterizing an excitation wavelength-dependent property. This can be ascribed to various surface states of C=O or amide group’s role as discrete exciton trapping centers to affect the emission energy in PL process [11, 19, 28]. Polydispersity and surface heterogeneity is the origin of excitation wavelength-dependent PL behavior [28, 29]. The broad absorption peak at around 430 nm is an ensemble of various surface states, including carboxyl and amide, which enables excitation wavelength-dependent PL behavior of N, S-CQDs. The fluorescence lifetime of N, S-CQDs was determined to evaluate its optical property (Fig. 2d). The PL decay curves of N, S-CQD sample can be fitted by a double-exponential formula, where *τ*_1_ is 3.48 ns, *τ*_2_ is 11.05 ns, and the average lifetime is 6.72 ns. Compared to the average lifetime of 2.42 ns of pristine CQDs [30], dramatic longer fluorescence lifetimes of both *τ*_1_ and *τ*_2_ were obtained on our sample. It has been reported that the *τ*_2_ proportion and average lifetime become longer with the N-doping amount increasing and concluded that the longer *τ*_2_ stemmed from the surface states [11, 31].

**Fig. 4:**
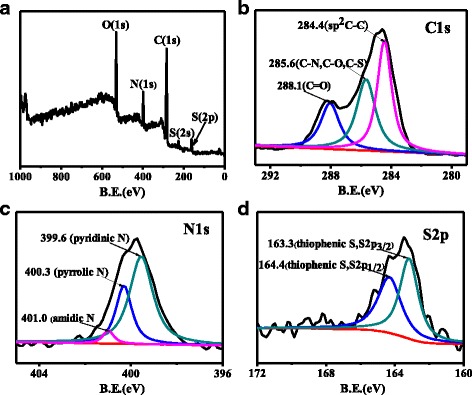
**a** The full scan XPS of N, S-CQDs. The high resolution XPS of C 1*s* **b** N 1*s* **c** and **d** S *2p* spectra of N, S-CQDs

The formation of N, S-CQDs was corroborated by XPS. As displayed in Fig. 4a, five distinct peaks at 530, 399, 284, 222, and 164 eV present of O 1*s*, N 1*s*, C 1s, S 2*s* and S 2*p* signals, respectively, indicating N and S were indeed injected into the framework of CQDs [17]. High-resolution C 1*s* XPS spectrum (Fig. 4b) indicates three characteristics of C structure, including aromatic conjugated *sp* ^2^ C (C=C) at 284.4 eV, *sp* ^3^ C (C–N, C–O, C–S) at 285.6 eV, and C=O/C=N at 288.1 eV [11]. The N 1*s* XPS spectrum (Fig. 4c) of the N, S-CQDs shows three peaks at 399.5, 400.3, and 401.0 eV, which represent pyridinic N, pyrrolic N, and amidic N, respectively [17, 24]. In the g-CNQD, the atomic ratio Ncore/Ccore, as derived from the experimental XPS intensity ratio, is equals to 1.40, which is close to the expected value of 1.33 expected for a C_3_N_4_ [19]. We conducted a similar data analysis of our sample, taking 285.6 eV of C 1*s* peak as “Ccore” while both 400.3 eV (pyrrolic) and 399.6 eV (pyridinic) of N 1*s* as “Ncore” (because the binding energy values are similar with 399.9 eV of NCore in the [19]), and the calculated Ncore/Ccore is 0.43, much smaller than 1.33 for C_3_N_4_. Moreover, the relative ratio of pyridinic N to pyrrolic N in our N, S-CQDs was found to be very different from that of N- or N, S co-doped CQDs synthesized by hydrothermal method [17, 21]. Pyridinic N is the dominant dopant in our N, S-CQD sample, which is 1.5 times as pyrrolic N, but it is usually less than 1.0 for many thermal synthesized samples. Such high pyridinic N may endow N, S-CQDs superior property for further catalysis application due to they can act as catalytic active sites [32]. Moreover, the pyrrolic N on the edge is an important composition of surface defects and can act as photoluminescence center [17, 27]. The S 2*p* XPS spectrum (Fig. 4d) displays two typical signals at 163.3 and 164.4 eV, which correspond to S 2*p*_3/2_ and S 2*p*_1/2_ of thiophenic S, respectively [16]. Combining with the FT-IR spectrum, we speculate that sulfur atoms successfully dope into the framework of N, S-CQDs as thiophenic S and exist at the edge of N, S-CQDs to improve their PLQY.

**Fig. 5:**
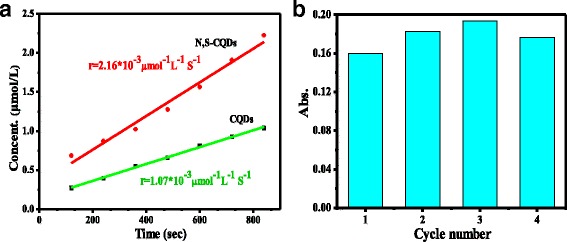
**a** The initial reaction rate of N, S-CQDs and free doped CQDs. **b** Reusability test of N, S- CQDs

Enzyme catalysis is expectative owing to its high specificity and activity. Horseradish peroxidase (HRP) is the most studied plant enzyme which contains the active center of porphyrin cycle in heme group to catalyze effectively the one electron oxidation, a wide variety of organic and inorganic substrate by hydrogen peroxide [33, 34]. The mimicking properties of pyridinic-rich N, S-CQDs were tested for the oxidation of the peroxidase substrates of 3, 3′, 5′, 5′-tetramethylbenzidine (TMB) in the presence of H_2_O_2_ by measuring the absorption of blue oxidation product of TMB at 652 nm. The UV/vis absorption of N, S-CQDs peaks at 234 and 340 nm. Figure 5a illustrates the fitting lines of the concentration of TMB-derived oxidation products (μmol/L) via time in the presence of N, S-CQDs and pristine CQDs. The reaction rate (*r*) for the decomposition of H_2_O_2_ on N, S-CQDs as enzyme mimics is 2.16 × 10^−3^ μmol^−1^ L^−1^ S^−1^, which is two times higher than that of pristine CQDs and previous reported doped-free CQDs under the same conditions [35, 36]. The excellent activity of pyridinic-rich N, S-CQDs can be attributed to the high doping content of N that possesses large electronegativity than carbon atom to increase the electron density of N, S-CQDs and, especially, the dominant pyridinic N owning one lone pair of electrons that leads to the enhancement in the electron density and mobility in the π-conjugated framework of N, S-CQDs, thus accelerates the reaction. This is the first report on the dramatic improving of catalase-mimic property of CQDs dependence on the dominant doping of pyridinic N in carbon framework.

The reusability of N, S-CQDs was investigated by consecutive four times usage for catalase-mimic reaction (Fig. 5b). On the four-cycle usage, no obvious decrease in the activity of N, S-CQDs was observed. The high stability of the intrinsic catalysis activity of N, S-CQDs is ascribed to the dominant pyridinic N-doping in the C=C framework because the pyridinic N can play a role as effective enzyme mimic catalytic sites for H_2_O_2_ decomposition.

## Conclusions

In summary, we synthesized a pyridinic-rich N, S-CQDs with high N- and S-doping level by microwave solid polymerization method within mere 50 s. Thiourea roles not only as S source but also as weak base to accelerate the intermolecular dehydration at low temperature and multistep carbonization, which enables the high N- and S-doping level in N, S-CQDs and dominant pyridinic N to inject into the conjugated framework as the enzyme mimic active sites. Our work provides an effective method to synthesize pyridinic-rich N, S-CQDs possessing both high PLQY and enzyme mimics activity.
